# Protective Effect of Silibinin on Lipopolysaccharide-Induced Inflammatory Responses in Equine Peripheral Blood Mononuclear Cells, an In Vitro Study

**DOI:** 10.3390/ani10112022

**Published:** 2020-11-03

**Authors:** Enrico Gugliandolo, Rosalia Crupi, Vito Biondi, Patrizia Licata, Salvatore Cuzzocrea, Annamaria Passantino

**Affiliations:** 1 Department of Chemical, Biological, Pharmaceutical and Environmental Science, University of Messina, 98166 Messina, Italy; egugliandolo@unime.it; 2 Department of Veterinary Science, University of Messina, 98168 Messina, Italy; rcrupi@unime.it (R.C.); vbiondi@unime.it (V.B.); plicata@unime.it (P.L.); passanna@unime.it (A.P.); 3 Department of Pharmacological and Physiological Science, Saint Louis University, School of Medicine, 1402 South Grand Blvd, St Louis, MO 63104, USA

**Keywords:** silibinin, horse, whole blood

## Abstract

**Simple Summary:**

Natural compounds are often an important source of biologically active molecules, which can find important applications in the treatment or pharmacological prevention of several pathologies. Silibinin is a natural polyphenolic flavonoid that is extracted from plant milk thistle, *Silybum marianum.* Silibinin has been reported to have antioxidant and immunomodulatory and anti-inflammatory activities. In horses, in particular, inflammation secondary to bacterial infection or translocation is one of the most frequent causes of morbidity and mortality. The aim of this study was to test the effect of silibinin on lipopolysaccharide (LPS)-induced inflammatory response in equine peripheral blood mononuclear cells (PBMCs). Taken together, our results showed an interesting prospective in therapeutic use of silibinin in equine inflammatory disease. Furthermore, the results from this study support the evidence of use equine PBMCs as an in vitro model to study inflammatory and immune response and for drug screening into the target specie.

**Abstract:**

Although inflammation is an important physiological response, it plays a prominent role in several diseases across the mammalian species. In horses, in particular, inflammation secondary to bacterial infection or translocation is one of the most frequent causes of morbidity and mortality. Research in new molecules with anti-inflammatory and immunomodulatory proprieties and safe use profile is constantly an active field; natural compounds are an important source of molecules with peculiar properties such as antioxidants, anti-inflammatory and immune modulating. Silibinin, a natural polyphenolic flavonoid, extracted from plant milk thistle, *Silybum marianum,* has been reported to have actions such as antioxidant immunomodulatory and anti-inflammatory. The aim of this study was to test the effect of silibinin on lipopolysaccharide (LPS)-induced inflammatory response in equine peripheral blood mononuclear cells (PBMCs). Our results showed the protective effect of silibinin 10 μM and 50 μM in equine PBMCs stimulated with LPS. Silibilinin was able to prevent the LPS induced increased levels of TNF-α, IL-1β, IL-6 and IL-8. The results from this study on LPS-stimulated equine PBMCs showed that silibinin could be a useful pharmacological approach in treatment or prevention of several inflammatory conditions in horse.

## 1. Introduction

Inflammation is widely known to be associated with several pathological conditions across different species. In particular, in equids, inflammation secondary to bacterial infection or translocation is one of the most frequent causes of morbidity and mortality [1]. Furthermore, inflammation is a key factor for several horse inflammatory diseases such as laminitis [2]. Inflammation is also responsible for a common and harmful condition in horses known as Systemic Inflammatory Response Syndrome (SIRS). SIRS, which constitutes a lifelong risk for horses, can occur during sepsis, infections (bacteria, fungi, viruses, etc.) or by non-infectious causes (toxins, acidosis, trauma, etc.) [2]. During septicaemia or the endotoxemia-induced inflammatory response, the immune response activation is a fundamental step, for the progression and worsening of these diseases [3]. Thus, the gold standard for an effective drug against these diseases such as SIRS must include an immune modulating and anti-inflammatory action. Nonsteroidal anti-inflammatory drugs (NSAIDs) are the most commonly used anti-inflammatory drugs in horse [4], although the widely recognised side effect and long term toxicity [5]. Also the introduction of COX-2 selective inhibitor, not reached the expected prospects, in terms of efficacy and safe use [6]. Another important drug used in horse for endotoxemia, SIRS or septicaemia is Polymyxin B an antibiotic drug. Although its nature of antibiotic Polymyxin B is able to counteract free endotoxins, but its use is strongly limited for nephrotoxic and neurotoxic effect [7]. Thus, research/studies in new molecules with anti-inflammatory and immunomodulatory proprieties and safe use profile is constantly an active field. Natural compounds or Phytocomplexes are an important source of molecules with peculiar properties such as antioxidants, anti-inflammatory and immune modulating. Silibinin, a natural polyphenolic flavonoid, is the major active constituent of Silymarin extracted from plant milk thistle, *Silybum Marianum*. Silymarin is a mixture of isomeric complexes of flavonoid and flavolignans. The main components of Silymarin are silibinin, isosilibin, silychristin, isosilychristin, silydianin, and silimonin [8]. Silymarin and Silibinin are widely known such as a strong antioxidant. This strong antioxidant activity is mainly related to free radical scavenger action, and to an improvement of cellular antioxidant defence such as glutathion (GSH) and superoxide dismutase (SOD) levels [9]. Interesting silymarin and silibinin showed also immunomodulatory effects [10]. In fact, different studies show the anti-inflammatory effect through the suppression of NF-κB signaling pathway and TNF-α inhibition [11,12,13]. Interestingly for these compounds were observed different immunomodulatory activities in a dose and time-dependent manner, in particular seem that low doses inhibit T-lymphocyte function while at high doses seems to act as stimulant, indicating an important role as immune response modifier [10]. Interesting recently has been reported the protective effect of silymarin on LPS-induced inflammation in the hoof dermal cells of dairy cows and in particular, the protective effect of silibinin on LPS-induced inflammation on the lamellar tissue [14,15]. Furthermore the safety and the pharmacokinetics of silibinin in horse has been demonstrated, emphasizing an excellent safety profile and limited oral bioavailability in horses, however given the limited amount of information about it, future studies will be needed to clarify these aspects [16]. Based on the knowledge mentioned above, our hypothesis was that silibinin has an anti-inflammatory and regulatory action on the immune response in equines. Then to test our hypothesis we used an ex vivo model challenging horse PBMCs with LPS in vitro.

## 2. Materials and Methods

### 2.1. Equine Donors and Blood Collection

Blood samples were obtained from 10 clinically healthy jumping horses (5 geldings and 5 mares; 7–14 years old; mean body weight: 500 ± 30 kg) with the informed consent of the owners. The health status of the horses was checked by physical examination, and the animals were free from internal and external parasites. No pharmacological treatment or nutraceutical supplementation was administered for 1 week prior to the study. All animals were managed equally and housed in individual stalls (3.5 × 3.5) at the same training centre located in Sicily (Italy) under natural photoperiod. Food (hay and a mixture of cereals) was provided three times daily, with water ad libitum. The blood (6 mL) was collected from all animals by jugular vein puncture into Vacutainer tubes with ethylenediamine tetra-acetic acid (EDTA) (Terumo Corporation, Tokyo, Japan) as anticoagulant. The protocols of animal husbandry and experimentation were performed in accordance with the standards recommended by the Guide for the Care and Use of Laboratory Animals and Directive 2010/63/EU for animal experiments.

### 2.2. Peripheral Blood Mononuclear Cell (PBMC) Isolation

Blood samples were 1:1 diluted in sterile phosphate buffered saline (PBS) containing 2 mM EDTA, and after layered over Ficoll^®^ Paque Plus (GE Healthcare), according to manufacturer protocols) PBMCs from the Ficoll layer were washed twice in PBS/EDTA. PBMCs were re-suspended in RPMI-1640 medium (Sigma-Aldrich, Milano, Italy) containing penicillin G (100 U/mL) (Sigma-Aldrich, Milano, Italy ), streptomycin (100 μg/mL) (Sigma-Aldrich, Milano, Italy), heparin (10 U/mL) (Sigma-Aldrich, Milano, Italy) and 10% horse serum, according on what has seen previously with slight modification [17].

### 2.3. Treatments and LPS Stimulation

PBMCs were seeded in 24 well plates at a density of 4 × 106 cells/mL and incubated for 2 h at 37 °C and 5% CO_2_. Where required cell was pretreated with Silibinin (Sil) (Sigma-Aldrich, Milano, Italy) for one hours before LPS stimulation 1 μg/mL (0111: B4, Sigma-Aldrich. Milano, Italy) for 4 h.

### 2.4. ELISA Assays

Cytokines levels were evaluated in supernatants x hours after LPS stimulation using Duo Set ELISA (R&D system, Minneapolis, MN, USA) for equine TNF-α, IL-6 and IL-10 according to manufacturer protocols [18].

### 2.5. RNA Isolation and RT-PCR Analysis

At the end of each time point for each experimental group, PBMCs were centrifuged, and supernatant was removed and stored for further analysis as seen above. Thus, RNA was isolated from PBMCs using RNeasy Mini Kit (Qiagen, Milan, Italy) according to manufacturer protocols. RNA was then quantified using a Nanodrop Spectrometer and subsequently an equal quantity of RNA for each sample used for cDNA synthesis using iScript^TM^ cDNA Synthesis Kit (Bio-Rad Milano, Italy) according to manufacturer protocol. iQ^TM^ SYBR Green Supermix (Bio-Rad, Italy). Real-time PCR was performed using a Bio-Rad CFX Real-Time PCR ((Bio-Rad Milano, Italy) Detection System, with specific designed equine primers as described previously [19,20]. Fold change in mRNA level was determined using the −ΔΔCt data analysis method [21].

### 2.6. Statistical Analysis

For each experiment, three independent experiments were performed and each experiment was used *n* = 10. The data resulting from all experiments are expressed as means ± SEM. Statistical differences between groups were compared using ANOVA, followed by Tukey’s test using GraphPad Prism version 8 (GraphPad Software Inc., La Jolla, CA, USA). A *p*-value of less than 0.05 was considered statistically significant.

## 3. Results

### 3.1. Silibinin Effect on Cytokines Production

**Figure 1:**
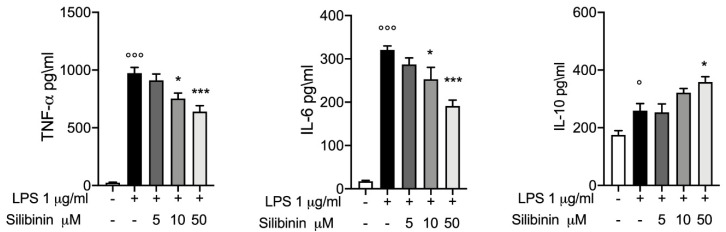
Silibinin effect on cytokines expressions ELISA assay for TNF-α, IL-6, IL-10, levels (pg/mL) in equine PBMCs treated or not with silibinin 5 μM, 10 μM and 50 μM for one hour before stimulation with LPS 1 μg/mL for four hours. Data are representative of at least three experiments, means ± SEM; ° *p* < 0.05 vs. control; °°° *p* < 0.001 vs. control; * *p* < 0.05 vs. LPS; *** *p* < 0.001 vs. LPS. (− absent; +present).

As showed in Figure 1, LPS 1 μg/mL simulation for four hours induces a significantly inflammatory response in equine PBMCs. In particular, Figure 1 Panel A showed that compare to control LPS stimulation induce a significative increase in TNF-α levels (24.81 ± 4.56 vs. 973.7 ± 48.92) one of the major pro inflammatory cytokines, the treatment with silibinin at dose of 5 μM for one hour before LPS stimulation produces no changes in TNF-α levels (910.6 ± 54.85). While the treatments with silibinin at 10 μM and 50 μM for one hour before LPS stimulation, showed a significantly protective effect in a dose-dependent manner (753.7 ± 46.80 and 639.9 ± 52.16 respectively). Then we evaluated IL-6 levels as a key regulator and driving factor in inflammatory response and in “cytokine storm”. Compared to control group, four hours post LPS stimulation in LPS group we found a significative increased level in IL-6 (17.22 ± 1.11 vs. 320.5 ± 9.117). Treatments with silibinin for one hour followed by LPS stimulation for four hours showed a dose dependent protective effect. Also, in this case the dose of 5 μM produces no significantly changes while the dose of 10 μM and 50 μM showed a dose dependent inhibition on LPS induced IL-6 increase (253.2 ± 27.36 and 191.1 ± 13.62 respectively). Finally, we evaluated the levels of IL-10 (immunoregulatory cytokine), and we observed an increased level in LPS group compared to control groups (175.1 ± 14.82 *vs.* 259.4 ± 24.36), and only silibinin at 50 μM significantly increase these trend (358.4 ± 18.74). 

### 3.2. Silibinin Effect on mRNA Cytokines Expressions

**Figure 2:**
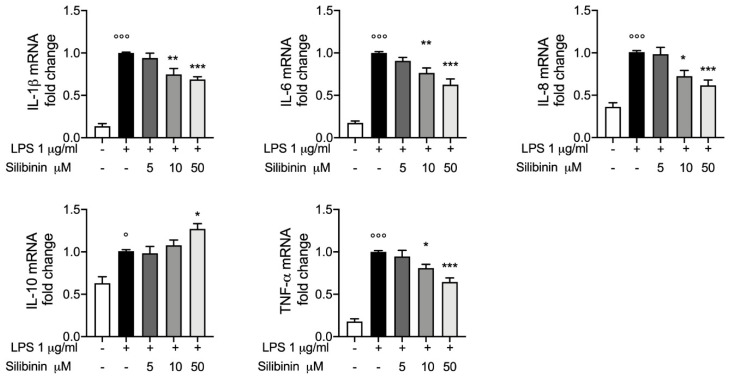
Silibinin effect on mRNA cytokines expressions RT-PCR assay for mRNA of IL-1β, IL-6, IL-8, IL-10 and TNF-α, levels express as mRNA fold change in equine PBMCs treated or not with silibinin 5 μM, 10 μM and 50 μM for one hour, before stimulation with LPS 1 μg/mL for four hours. Data are representative of at least three experiments, means ± SEM; ° *p* < 0.05 vs. control; °°° *p* < 0.001 vs. control; * *p* < 0.05 vs. LPS; ** *p* < 0.01 vs. LPS; *** *p* < 0.001 vs. LPS; − absent; + present.

To further investigate the effect of LPS exposure in equine PBMCs and the protective effect of silibinin, we move to evaluate the mRNA levels in different experimental conditions. As showed in Figure 2 according to ELISA results, we found that LPS 1 μg/mL for four hours induce a significative inflammatory response, in fact we found that TNF-α and IL-6 mRNA were significantly higher in LPS group than control group. Furthermore, we also evaluated IL-1β and IL-8 mRNA levels as key mediator in orchestrating the inflammatory and immune response, according on what has seen above we found significantly increase in IL-1β and IL-8 mRNA levels in LPS group compared to control group. RT-PCR result confirm the protective effect of silibinin treatment as seen for cytokines levels, in fact groups treated with silibinin at 10 μM and 50 μM showed a significantly reduction in IL-1β, IL-6, IL-8 and TNF-α mRNA levels compared to LPS group, and in particular these effect were observed in a dose dependent manner. For silibinin 5 μM treatment we observe no significantly changes compared to LPS. By RT-PCR we also confirmed the IL-10 mRNA levels, in according to ELISA assay for IL-10 interesting we observed a significantly increase IL-10 mRNA levels four hours post-LPS stimulation in group treated with silibinin at 50 μM.

## 4. Discussion

Although inflammation is an important physiological response, it also plays a prominent role in several diseases across the mammalian species. In horses, an exacerbate inflammatory response may lead a continuum of events that ranging from acute phase responses, SIRS to generalised immunosuppression [22]. Furthermore inflammation play a key role in harmful horse diseases such as laminitis [23], SIRS, sepsis, endotoxemia, colic, etc. [2]. Pathogen-associated molecular patterns (PAMPs) and damage-associated molecular patterns (DAMPs) are key molecules involved in the immune response activation trough the pattern recognition receptors (PRRs). Among the PRRs the Toll-like receptors (TLRs) play a central role in host cell recognition and responses to microbial pathogens and several stimuli [24,25]. TLR-4 play a key role in inflammatory response as is responsible for LPS recognition. In horses, TLR-4 has been seen to be able to interact with endotoxins and mediated inflammatory response, interestingly this effect has been seen to be greater in equine monocytes than in other species [26]. Several evidences suggest that as in other species, also in equids cytokines are the key mediators in inflammatory and immune response [27]. Nonsteroidal anti-inflammatory drugs NSAIDs, glucocorticoids and Polymyxin B is the first line of drugs choice, but their therapeutics application is limited due to important side effect [4,5,28]. Thus, an effective and more safety drugs is an important prospective for new pharmacological approach and preventive medicine therapy for inflammatory diseases. Thus, based on the above information, the aim of this study was to evaluate the protective effect of silibinin on LPS induced inflammatory response in equine peripheral blood mononuclear cells (PBMC). We chose to use this in vitro model of equine PBMCs as a readily available representative ex vivo model of immune cells in this animal species. Moreover equine PBMCs are important representative immune cell categories that play a key role in immune and inflammatory response [29,30]. Silibilin a polyphenolic flavonoid, is the major active component of of Silymarin extracted from plant milk thistle. Silimarin and its major active compound silibinin are known to have different biological properties, for example it is widely used in different liver disorders such as in chronic liver diseases, cirrhosis and hepatocellular carcinoma, because of its antioxidant, anti-inflammatory and antifibrotic power [12]. A recent study highlighted the potential therapeutic effect of silibinin for the treatment of equine laminitis, and demonstrating the inhibitory effect on reactive oxygen species (ROS) production and myeloperoxidase (MPO) release by stimulated equine neutrophils (PMNs) and on MPO activity [31]. Moreover, silibinin has been shown to produce anti-inflammatory and immune modulating effects through inhibition of Nf-κB [32]. In this study, to test the anti-inflammatory and immune response modifier activity of silibinin, we choose an ex vivo model of PBMC stimulation. As inflammatory stimuli in PBMC the stimulation with LPS 1 μg/mL for four hours, previously seen [19,20] and the treatments with silibinin were performed one hour before LPS stimulation, with the concentration of 5, 10 and 50 μM. Although these treatment timings do not reflect the clinical situation, this method is widely validated and necessary, for a better evaluation of cytokine secretion response [33] Several studies evidence the detrimental role of TNF-α in equine sepsis, colic and endotoxaemia [34] and correlate high level of TNF-α with a poor outcome or as well high mortality [35]. Ours results showed that four hours after LPS stimulation PBMC showed a significantly increase in TNF-α. While group treated with silibinin 10 and 50 μM showed a significantly protective effect in a dose dependent manner on both TNF-α levels and mRNA. TNF-α signalling also induces the increase in IL-1β expression, together elevated levels of TNF-α and IL-1β is a key feature of equine sepsis, and poor prognosis [27]. In accordance with the results observed for TNF-α we observed after LPS stimulation a significantly increase in IL-1β as indicated by IL-1β mRNA levels. Also, in this case group treated with 10 and 50 μM showed a significantly reduced in IL-1β mRNA levels in a dose dependent manner. Then we move to evaluate IL-6 secretion and mRNA levels, although IL-6 does not play a prominent role as proinflammatory mediators it play a key role in immune response [36], and interesting like in human in horse high levels of IL-6 has been observed in non-surviving septic foals [37]. We evaluated IL-6 for both secretion and mRNA levels in PBMC after LPS stimulation, and according to an inflammatory response induced by LPS, we observed a significantly increased levels and mRNA for IL-6. The treatment with silibinin at 5 μM produces no significantly changes while group treated with 10 and 50 μM showed a significantly protective effect in a dose dependent manner on both IL-6 levels and mRNA. Regarding immune response another important mediators for immune response orchestration is the IL-8 [38]. We observed after LPS stimulation a significantly increase in IL-8 mRNA levels. While group treated with 10 and 50 μM showed a significantly reduced IL-8 mRNA levels in a dose dependent manner. On the other hand there are several mediators that counterbalance the proinflammatory response and act as immunomodulatory signals, the most commonly anti-inflammatory and immunomodulating mediator is IL-10 [39]. After LPS stimulation equine PBMCs showed a moderate increase in IL-10 levels and mRNA, while the treatment with silibinin at a dose of 50 μM showed an increased level and mRNA of IL-10 compared to LPS.

## 5. Conclusions

Taken together, our results showed an interesting prospective in the use of silibinin in inflammatory disease in horses. Furthermore, the results from this study support the evidence of use equine PBMCs as an in vitro model to study inflammatory and immune response and for drug screening in the target specie. Although, further study is required to confirm the therapeutic role of silibinin in horse the mode of action for silibinin demonstrated in this study on LPS stimulated equine PBMCs showed that silibinin could be a useful pharmacological approach in treatment or prevention of several inflammatory conditions in horse.
